# Creating Effective, Evidence-Based Video Communication of Public Health Science (COVCOM Study): Protocol for a Sequential Mixed Methods Effect Study

**DOI:** 10.2196/34275

**Published:** 2022-03-11

**Authors:** Jo Røislien, Jane K O'Hara, Ionica Smeets, Kolbjørn Brønnick, Siv Hilde Berg, Marie Therese Shortt, Daniel Adrian Lungu, Henriette Thune, Siri Wiig

**Affiliations:** 1 SHARE - Centre for Resilience in Healthcare Faculty of Health Sciences University of Stavanger Stavanger Norway; 2 School of Healthcare Faculty of Medicine & Health University of Leeds Leeds United Kingdom; 3 Science Communication and Society Institute of Biology Leiden University Leiden Netherlands

**Keywords:** pandemics, risk, public health, science communication, mixed methods, evidence-based medicine, COVID-19

## Abstract

**Background:**

The nonlinear nature of contagious diseases and the potential for exponential growth can be difficult to grasp for the general public. This has strong implications for public health communication, which needs to be both easily accessible and efficient. A pandemic is an extreme situation, and the accompanying strict societal measures are generally easier to accept if one understands the underlying reasoning behind them. Bringing about informed attitude change and achieving compliance to strict restrictions requires explanations of scientific concepts and terminologies that laypersons can understand.

**Objective:**

The aim of the project is to develop effective, evidence-based modes of video communication for translating complex, but important, health messages about pandemics to both the general population and decision makers. The study uses COVID-19 as a case to learn and prepare society for handling the ongoing and future pandemics, as well as to provide evidence-based tools for the science communication toolbox.

**Methods:**

The project applies a mixed methods design, combining qualitative methods (eg, interviews, observational studies, literature reviews) and quantitative methods (eg, randomized controlled trials [RCTs]). The project brings together researchers from a wide range of academic fields, as well as communication industry professionals.

**Results:**

This study has received funding from the Trond Mohn Foundation through the Research Council of Norway’s “COVID-19 Emergency Call for Proposals” March 2020. Recruitment and data collection for the exploratory first phase of the project ran from February 2021 to March 2021. Creative communication work started in May 2021, and the production of videos for use in the RCTs in the final phase of the project started in September 2021.

**Conclusions:**

The COVCOM project will take on several grand challenges within the field of communicating science and provide evidence-based tools to the science communication toolbox. A long-term goal of the project is to contribute to the creation of a more resilient health care system by developing communication responses tailormade for different audiences, preparing society for any future pandemic.

**International Registered Report Identifier (IRRID):**

DERR1-10.2196/34275

## Introduction

A pandemic is an extreme situation, and extreme measures are needed to combat it. Although similar numbers of people die annually in traffic or from cancer, the number of people who catch a contagious disease can grow at a rapidly increasing rate. This nonlinear nature of contagious diseases and the potential for exponential growth can be difficult to grasp for the general public. At the same time, this feature of contagious diseases brings about a need for measures that are not only rapid but also often radical. This has strong implications for public health communication, which needs to be both easily accessible and efficient. 

During the COVID-19 pandemic, scientific knowledge from various fields of health research has been at the heart of decision-making. Strong preventive and societal measures—with substantial implications on peoples’ lives—have been central to combating the pandemic. Such measures are generally easier to accept if one understands the underlying reasoning behind them [1-3], and bringing about informed attitude change and achieving compliance to strict restrictions thus require explanations of scientific concepts and terminologies that laypersons can understand. Therefore, in order to effectively tackle pandemics, the provision of large-scale information outreach is imperative.

However, how to do this—or how *not* to do it—is poorly understood, with several grand challenges, sparse research within the field, and limited available empirical evidence for making generalizations [4,5]. Systematic reviews [6-9] have found multiple knowledge gaps in pandemic risk communication, for example how risk perception is framed by competing narratives, the lack of in-depth learning from past experiences [8], and slim evidence of the effectiveness of pandemic risk communication [6].

Guaranteeing the flow of information to engage stakeholders is important [9]. Different stakeholders (eg, policy makers, infection control bodies, primary and specialized health care providers) need a common understanding of risk, risk implications, and consequences. This implies that the understanding of key scientific information, and the lack thereof, might facilitate understanding of why national and international societies have not been able to learn from the past and prepare for predicted catastrophic pandemics like COVID-19 [10].

Although crisis communication is an established research field within leadership [11], little is known about the communication of the science upon which leadership and decision-making are based. In the COVID-19 pandemic, this typically entails health science topics related to contagious diseases, including mathematics, epidemiology, risk, medicine, and health. Establishing evidence-based communication that helps facilitate understanding, attitudes, and risk behaviors, and what works for whom, is essential.

The first empirical gap analysis in the field of science communication—including ~3000 papers—identified several grand challenges [4]. First, the field is mostly limited to one-off studies, and there is a need for longitudinal, experimental, comparative, and wider systemic research to understand how contents and channels, actors, and audiences interrelate. Second, the field is caught in disciplinary structures, and the opportunity for interdisciplinary integration has not been seized. Third, there is a lack of transfer and collaboration between researchers and practitioners, and getting practitioners involved in the research has been suggested to bridge the divide. Randomized controlled trials (RCTs) in science communication are few, with retrospective analyses being the norm. The move toward evidence-based science communication, inspired by the impact of evidence-based medicine over the past decades, has been called for [5]. Experimental studies, capable of invalidating previously accepted practices and replacing them with new ones that are more accurate and effective [12], is an integral part of achieving this.

All communication takes place within a continuously changing culture, and narratives, images, and metaphors that worked yesterday might not work today. This must be taken into account when creating health science communication. *How* you say something is as important as *what* you say. Arts is central for outreach [13], and in March 2020, the World Health Organization (WHO) sent out an open global call to creatives to help with communication in the COVID-19 pandemic [14,15]: Creativity in health science communication is a necessity. It is however poorly understood and underused.

The population’s media habits have changed rapidly in recent years. Driven by social media and smartphones, each citizen can now choose on what to spend their time. Video consumption is increasing, and video makes up an estimated 80% of all internet traffic [16]. YouTube, the world’s largest video site, has 2 billion monthly users, with >500 hours of content uploaded every minute [17]. In the United States, YouTube on mobile alone reaches more 18- to 49-year-olds than any cable TV network [18]. However, despite the scale of video consumption, there is little extant evidence to guide the effective use of video for relaying complex health messages. It is well-known in risk and public health communication that trust is key [19,20], and there is a growing body of research on narrative-based methods and digital storytelling within medicine and health [21,22]. However, much research remains before we fully understand how to best utilize the video format for effective public health communication.

Film is a powerful communication format, frequently used in advertising and popular culture. It is a collective process in which screenwriters, directors, cinematographers, set designers, and professional performers come together to tell a story using moving pictures. Artistic choices are central to filmmaking but are rarely considered when communicating science through film. In an analysis of 400 science videos on YouTube, videos generally fell into 1 of 4 categories: video-blog, voice-over animation, recorded presentation, or interview [23]. Notably, none of the most viewed videos on YouTube in 2019 fell into any of these 4 categories. That is, the video styles most often used by science communicators are not the video styles that tend to attract large audiences, indicating that there is something to be gained by exploring other creative means when producing videos in order to attract larger—and other—audiences when communicating scientific knowledge. In order to create effective science communication, interdisciplinary collaboration of experts from disciplines with different norms and practices is needed [24]. Achieving this is challenging, and the field remains immature.

To tackle the grand challenges within the field of science communication [5], the current project takes an interdisciplinary approach to developing effective communication for pandemics. Creating effective science communication requires collaboration between not only scientists with expert knowledge in the subject matter and in communication [25] but also actual communicators. This study brings together experts from a wide range of fields, including researchers from health studies, humanities, risk, societal safety, medicine, nursing, public health studies, psychology, visual communication, epidemiology and statistics, as well as mass media professionals, communicators, and filmmakers, in order to ensure first-hand cultural know-how for contemporary communication. This is done order to move from experience to evidence-based communication.

The primary objective of the study is to use video to develop effective, evidence-based modes of communication for translating complex, but important, health messages about pandemics. The study uses COVID-19 as a case to learn and prepare society for handling the ongoing and future pandemics, as well as to provide evidence-based tools for the science communication toolbox. This will be achieved through the following secondary objectives:

1. Identify communication strategies and key topics about pandemics that public health scientists and officials need to communicate
2. Explore communication strategies and artistic dimensions in filmmaking for the creation of effective science communication videos aimed at lay viewers, focusing primarily on the adult part of the Norwegian speaking population with a general primary school level understanding of science
3. Test the effect of these videos through controlled experiments, coupling communication to learning outcome and individual differences such as attitude(s) and compliance towards the topic(s), exploring also sociodemographic variables
4. Identify the features of the most effective videos on a mass communication scale in collaboration with national mass media broadcasters

We hypothesize that shorter, props-driven videos will outperform longer, more static and scientifically focused videos in terms of comprehension of the topics communicated, while trust will be higher in the latter. For intensions, behavior, and behavior change, we hypothesize that narratives and metaphors will be more effective than the factual scientific information itself. Further, optimizing communication for accessibility by reducing scientific precision will not significantly reduce comprehension, intensions, or active behavior. Finally, observed effects will vary according to the demographic characteristics of the receiver.

## Methods

### Study Design

The study applies a sequential mixed methods design [26], combining various qualitative and quantitative research methods.

**Figure 1:**
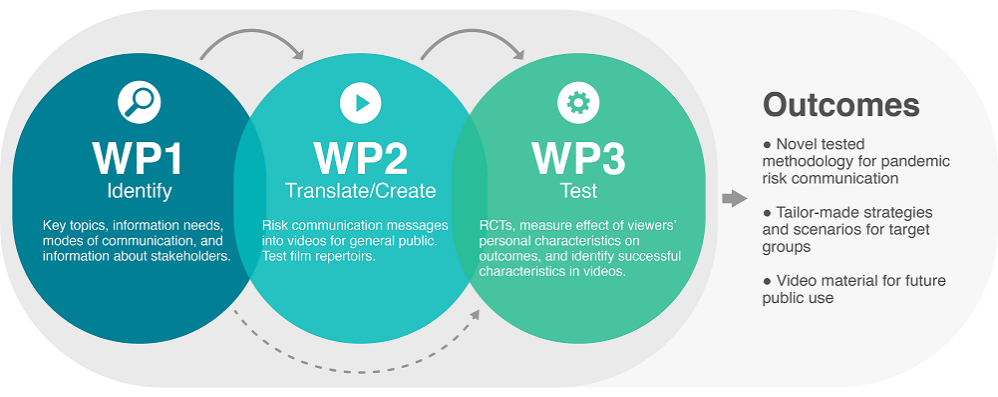
Study design and workflow of work packages (WPs). RCTs: randomized controlled trials.

Research in science education [27,28], health communication [29], and cognitive anthropology and psychology [30,31] suggests that people interpret new information in light of their existing beliefs. Rather than relying on scientists’ opinions only, the communication of scientific knowledge should thus be based on evidence of the audience’s relevant beliefs and what they are still missing [32,33]. Aligned with this, we apply a so-called mental models approach to developing communications as our theoretical anchor for the sequential design to communicating scientific knowledge [34]: First, identify what people need to know to make more informed decisions; second, identify what they already know and how they make decisions; third, create the communication; fourth, test its effectiveness. This approach resonates with the call for an evidence-based approach to communicating science [5,35]. Correspondingly, the project is operationalized into 3 work packages (WPs) that build on each other (Figure 1). These 3 phases of the project are described in detail in the following sections.

### Recruitment

A large part of the study will run throughout the course of a worldwide pandemic, with the accompanying societal restrictions setting the context of the project. Most of the data collection will take place digitally, with reduced need for travel and physical meetings, using online surveys, video interviews, and interactive methods (eg, stakeholder analyses and workshops). The study population will include both experts and representatives from the general public as part of a holistic approach to how health risk and consequences are understood and communicated on all levels. More detail about the individual WPs is provided in the following sections.

### Phase 1: Establishing What Needs to Be Communicated (WP1)

WP1 will apply multiple qualitative methods to identify communication strategies and key topics related to pandemic risks to be communicated to the public. The views from both communicators and receivers of pandemic information are included. WP1 will inform WP2 and WP3 on what people already know, how they make decisions, and what they need to know to make more informed decisions when facing pandemic risks [32,34].

#### Rapid Scoping Review

The WP will involve a rapid scoping review [36,37] to obtain an overview of the evidence pertaining to diverse modes of communication used by health authorities in health risk communication with the public during a pandemic. The databases MEDLINE and EMBASE will be searched for publications from 2009 to 2020. This will provide information about key scientific concepts, types of outcomes, and research gaps related to diverse modes of communication.

#### Mental Models

Employing semistructured individual interviews, WP1 will involve the creation of 2 mental models. First, a public mental model will explore how the public perceives difficult scientific concepts and acts on the public health risk communication related to COVID-19. Second, an expert mental model will identify key topics and communication strategies related to pandemic risk [32]. Analyses will be guided by the mental model framework by de Bruin and Bostrom [34]. Mental models are representations of how something works in the real world, and the framework by de Bruin and Bostrom [34] was developed to assess what to address in science communication.

Rather than recruiting a large representative sample, it is recommended to recruit a small but diverse sample of participants (~10-15 participants) when exploring mental models [34]. For the public mental model, we apply a purposive sample of Norwegian citizens between 18 years and 80 years old, with various levels of education, gender, and a range of geographic regions in Norway. For the expert mental model, we include experts with various levels of knowledge of issues regarding COVID-19 risk and mitigation at different system levels (eg, municipality, hospital, national level, research). The identified topics will be analyzed in a directed content analysis, focusing on scientific concepts for public risk communication and the identification of possible new scientific concepts for pandemic risk communication [38].

#### Stakeholder Analysis

Finally, in WP1, we will undertake a stakeholder analysis to identify various stakeholder groups, assessing their different roles and values [39]. This to gain insight into the responsibility of different stakeholder groups, the perspectives of those involved in public health risk communication, and possible trade-offs in public risk communication. Here, we define stakeholders as individuals who represent a unit or organization that participates in public risk communication related to COVID-19 in Norway.

First, we will identify key stakeholder groups for pandemic risk communication at the local municipal level, at the hospital level, and at the policy level by reviewing publicly available policy documents describing the stakeholder’s roles and responsibility in pandemic management. Our sampling strategy is to include stakeholders with key roles in risk communication across these levels in the health care system. We then assess their different perspectives and values by interviewing a sample of ~10 stakeholders involved in communication of pandemic risk information representing different system levels. The document and participant interview data will be analyzed in a directed qualitative content analysis with predetermined categories for micro, macro, and meso levels and inductive category development to analyze values and perspectives [40]. Here, micro-level research refers to the examination of individuals and individual-level interactions (eg, intentions, feelings, and beliefs), the meso-level examines groups (including teams, units, and organizations), and macro-level research examines the political-administrative environment (including national systems, regulation, and cultures).

#### Recruitment and Sampling

User representatives, nongovernmental organizations, partners in the consortium from Stavanger University Hospital, Haukeland University Hospital, and the Center for Developing Institutional and Home Care Services Sogn and Fjordane will contribute to the recruitment in the various WP1 substudies. Participants will also be recruited by inviting eligible participants identified by the COVCOM team.

### Phase 2: Creating Communication (WP2)

Creative choices are central to filmmaking but are rarely considered when communicating science. In this WP, we will study the creative processes that underlie effective translation of scientific information into understandable communication.

#### Video Review

To explore how videos created by health authorities measure up to contemporary video content, WP2 will include a video review of existing COVID-19 video communication. Online sites for Norwegian health authorities, including health entities at both national and regional levels, will be searched for video content, and entities with a dedicated YouTube channel featuring COVID-19–related video content will be included. These videos will be compared with COVID-19–related videos created by the WHO, as well as the most watched COVID-19 videos on YouTube. Aiming for a comparable number of videos, we will select, for example, the top 10 to 20 videos on YouTube for each of the search terms “covid 19” and “corona virus” using the YouTube search engine. Press briefings, live videos, and news reports will be excluded: We are interested in purposely produced video content rather than mere recordings of ongoing events. A content analysis of video formats and creative means utilized will be carried out to identify how health authorities measure up to contemporary video communication, both creatively and in reaching video consumers, identifying potential shortcomings and potential for improvement.

#### The Communicators’ Views

Aiming to uncover structural differences and similarities in how communicators from different fields approach the creation of health science communication, we will recruit 2 participants from each of 6 different professions: public health communication, health communication, science TV and film production, video journalism, creative advertising, and social media. First, we will conduct semistructured individual interviews, identifying their approaches to video communication, their thoughts on existing COVID-19 videos, and their take on interdisciplinary collaboration. Participants will then be paired profession-wise and observed while discussing ideas for new pandemic-related video communication. Through content analysis gaps and differences in the weighting, sequencing and overall approach when tasked with creating pandemic video communication will be identified.

#### Turning Scientific Information Into Accessible Videos

Informed by WP1, professional audiovisual science communicators will create videos with explanations of key topics related to pandemics aimed at laypersons. This entails a focus on factors like choice of sender, ethos of the messenger, semantics, properties of context, metaphors, use of props, visual language, cinematic techniques, and editing. The output will be multiple research-based videos developed to translate pandemic scientific information in different ways, ready for experimental testing and assessment of the effect of creative choices on outcomes related to learning and attitudes toward the topic (in WP3).

This creative work will be observed, with an estimated 10 group sessions involving 1 hour of creative concept development and 10 hours of observation of the production, supplemented with 10 half-hour individual interviews. These time estimates are pragmatically chosen as estimates of how much—and how closely—the creative process and the production should be followed in order to gain sufficient insight into the process. These times are flexible and can be adjusted if needed.

Following the full video production throughout the WP will help untangle the creative process as performed by industry professionals when developing effective health science communication. The study will be guided by the creative process stages (CPS) model with its 17 stages that make up the artistic creative process [41]. This enables the study of the creative process at the macro level (the main stages) and micro level (the thinking underlying the stages) and takes an ecological approach: observation in the natural environment while the creative work is unfolding. This enables a direct, rich, and inexpensive assessment of the creative process, and the method has high ecological validity [41]. Based on the CPS model, WP2 will involve deductive development of a methodology for visualizing creative processes, so as to be able to capture, analyze, and visually present the multidimensional and intertwined aspects and stages involved in creative communication work.

#### Recruitment and Sampling

Participants will be recruited through the project’s consortium members and its wider network, as well as through project members’ own networks, including Stavanger University Hospital, Center for Developing Institutional and Home Care Services Sogn and Fjordane, Norwegian Institute of Public Health, Dagbladet, Anorak, Bulldozer Film, and Nordic Screens. Gender balance will be taken into account in the recruitment process, along with age, educational background, and occupation. All interviews and observation studies will be carried out virtually using Zoom.

### Phase 3: Evaluating the Communication’s Effect (WP3)

Building on the work in WP1 and WP2, WP3 will involve quantitative experiments to assess the effect of various factors related to audiovisual public communication of health science in general and pandemics in particular.

#### Scoping Review

First, in WP3, we will conduct a scoping review focusing on the recipients of the communication. We will search 3 main databases for public health, social sciences, and biomedical studies: PubMed, Scopus, and Embase. The search string will be designed based on the PCC (Population-Concept-Context) framework as recommended by the Johanna Briggs Institute Manual for Evidence Synthesis and scoping reviews [42] as a less restrictive alternative to the PICO (population, intervention, comparator, and outcome) mnemonic recommended for systematic reviews. The scoping review is expected to provide a comprehensive view of the state of the art and give insight about characteristics of recipients and how they can impact the outcomes of health video communication. The knowledge will contribute to the interpretation of findings from the WP’s experimental studies described in the following sections.

#### Randomized Controlled Trials

In WP3, we will perform 2 RCTs with a factorial between-subjects experimental design including various factors related to the video communication. The first RCT will assess nonvisual factors in health communication videos such as the effect of the video super of the presenter (ie, whether the person on screen is labeled as “Professor” or “Citizen” in the text at the bottom of the screen) or whether the message presented is neutral or includes a “call to action” (ie, whether the presenter merely delivers factual information or also encourages the viewer to act). The final choice of nonvisual factors will be informed by work in the preliminary phases of the project. Assessing the effect of such nonvisual factors will be done by having an actor recite different scripts to the camera. The second RCT will use the videos developed by professional video creators in WP2.

The videos will be screened for representativeness of the population. After consent, participants will be randomized to different videos and between-factors conditions. A link will lead them to a web page where the procedure is explained. For the first RCT, this web page differs according to ascribed Source and Messenger, Content, and Engaging factors. Participants will then be shown the video(s), followed by the questionnaires. Here, we focus not only on learning outcomes and the understanding of risk at a conceptual level but also on attitudes toward the subject, behavioral change, and compliance with the message. Gender, cultural background, age, education, and work variables will be entered as covariates in the statistical analyses and in targeted subgroup analyses.

All data collection in WP3 will be conducted online, using online surveys (Survey Monkey) and collaborative tools. With a fully digital and completely automated random-number generator and allocation procedure, video link submission and measurement instruments can be performed automatically, keeping the process free from experimenter input.

Both RCTs foresee the possibility of a longitudinal study through a follow-up survey aiming to assess whether communication outcomes (eg, learning, attitudes) persist over time, evaluate the motivation of participants to rejoin the study based on the video alternative they have been exposed to, and explore whether communication outcomes are influenced by the evolution of the COVID-19 pandemic (eg, vaccination coverage, recent infection trends).

#### Real-Life Observational Study

Based on the RCTs, the videos used in the experiments will go through mild editing and then be screened for audiences on a mass scale, tracking spread and exploring engagement metrics such as likes and comments. This will be done in collaboration with consortium partners (eg, the national newspaper, Dagbladet, and video influencer network, Nordic Screens). Here, success criteria will be assessed through strategies such as tracking of how much of a video the viewers watch before skipping, likes, sharing, and response rates to “click here for more” links and coupled with demographic data.

#### Recruitment and Sampling

Participants for the RCTs will be recruited through invites from consortium partners such as the Norwegian Air Ambulance Foundation (NAAF). Statistical power depends on a number of factors. For the first RCT, a power analysis in G*Power [43] assuming a medium effect-size (f^2^≥0.25) and a full factorial design with 5 covariates in an analysis of covariance (ANCOVA) yields a conservative sample size of 401 to achieve a statistical power of 95%. For the second RCT, a smaller sample size is needed, as there are fewer covariates. The NAAF database of financial supporters consists of approximately 150,000 people from the general public, and recruitment of only a small fraction of them (1%) will thus suffice for the intended power.

### Ethical Considerations

The study is approved by the Norwegian Centre for Research Data (WP1 Ref number 583192, WP2 Ref number 703372) and exempted from ethical approval from the Regional Ethical Committee.

## Results

The COVCOM study is supported by the Trond Mohn Foundation (TMS) grant number TMS2020TMT10 and the University of Stavanger, Norway.

Recruitment and interviews for Phase 1 of the project ran from February 2021 to March 2021. Creative communication work in Phase 2 started in May 2021, and video production for use in the RCTs started in September 2021. Preparation for the RCTs in Phase 3 started in January 2021, with recruitment and data collection planned for 2021/2022.

## Discussion

The COVCOM project will take on several grand challenges within the field of communicating science and provide evidence-based tools to the science communication toolbox. A long-term goal of the project is to contribute to the creation of a more resilient health care system by developing tailor-made communication responses for different audiences, preparing society for any future pandemic [44].

The different backgrounds and perspectives of the individuals in the COVCOM project operate along different axes in terms of norms and what constitutes a project’s success. Getting all involved individuals to pull in the same direction makes the project challenging. The serial structure of the WPs, where unexpected results and delays in one WP might affect other WPs, puts high demand on collaborative efforts in the group. Clear communication and close follow-up between WP leaders and other key personnel are central.

As the project has expected outcomes beyond the scientific community for stakeholders in terms of communicators (eg, Norwegian Institute of Public Health, Norwegian Directorate of Health, and the government) as well as recipients (eg, the general population, hospitals, and representatives of vulnerable groups) of health information, dissemination will not only be through academic publications but also focus on communicating research results to communication practitioners, health professionals, and the general public in adequate trade magazines, newspapers, and online fora. Popular science articles to disseminate results beyond academia will be encouraged and facilitated by the project manager.

## Abbreviations

ANCOVA: analysis of covariance
COVCOM: COVID communication – Fighting a pandemic through translating science
CPS: creative process stages
NAAF: Norwegian Air Ambulance Foundation
PCC: Population-Concept-Context
PICO: population, intervention, comparator, and outcome
RCT: randomized controlled trial
TMS: Trond Mohn Foundation
WHO: World Health Organization
WP: work package
